# Further corroboration of distinct functional features in *SCN2A* variants causing intellectual disability or epileptic phenotypes

**DOI:** 10.1186/s10020-019-0073-6

**Published:** 2019-02-27

**Authors:** Anaïs Begemann, Mario A. Acuña, Markus Zweier, Marie Vincent, Katharina Steindl, Ruxandra Bachmann-Gagescu, Annette Hackenberg, Lucia Abela, Barbara Plecko, Judith Kroell-Seger, Alessandra Baumer, Kazuhiro Yamakawa, Yushi Inoue, Reza Asadollahi, Heinrich Sticht, Hanns Ulrich Zeilhofer, Anita Rauch

**Affiliations:** 1 Institute of Medical Genetics, University of Zurich, 8952 Schlieren, Zurich Switzerland; 2 radiz—Rare Disease Initiative Zürich, Clinical Research Priority Program for Rare Diseases, University of Zurich, 8006 Zurich, Switzerland; 3 Institute of Pharmacology and Toxicology, University of Zurich, 8057 Zurich, Switzerland; 4 Service de génétique médicale, CHU Nantes, 44093 Nantes, France; 5 Division of Child Neurology, University Children’s Hospital Zurich, 8032 Zurich, Switzerland; 6 Division of General Pediatrics, Department of Pediatrics and Adolescent Medicine, Medical University of Graz, 8036 Graz, Austria; 7 Children’s department, Swiss Epilepsy Centre, Clinic Lengg, 8008 Zurich, Switzerland; 8 Laboratory for Neurogenetics, RIKEN Center for Brain Science, Wako-shi, Saitama, 351-0198 Japan; 9 National Epilepsy Center, NHO Shizuoka Institute of Epilepsy and Neurological Disorders, Shizuoka, 420-8688 Japan; 10 Institute of Biochemistry, Friedrich-Alexander-Universität Erlangen-Nürnberg (FAU), 91054 Erlangen, Germany; 11 Institute of Pharmaceutical Sciences, ETH Zurich, 8093 Zürich, Switzerland; 12 Neuroscience Center Zurich, University of Zurich and ETH Zurich, 8057 Zurich, Switzerland; 13 Zurich Center for Integrative Human Physiology, University of Zurich, 8057 Zurich, Switzerland

**Keywords:** *SCN2A*, Nav1.2, Channelopathy, Patch-clamp, Epilepsy, Epileptic encephalopathy, Intellectual disability, Structural modelling, Electrophysiology

## Abstract

**Background:**

Deleterious variants in the voltage-gated sodium channel type 2 (Na_v_1.2) lead to a broad spectrum of phenotypes ranging from benign familial neonatal-infantile epilepsy (BFNIE), severe developmental and epileptic encephalopathy (DEE) and intellectual disability (ID) to autism spectrum disorders (ASD). Yet, the underlying mechanisms are still incompletely understood.

**Methods:**

To further elucidate the genotype-phenotype correlation of *SCN2A* variants we investigated the functional effects of six variants representing the phenotypic spectrum by whole-cell patch-clamp studies in transfected HEK293T cells and in-silico structural modeling.

**Results:**

The two variants p.L1342P and p.E1803G detected in patients with early onset epileptic encephalopathy (EE) showed profound and complex changes in channel gating, whereas the BFNIE variant p.L1563V exhibited only a small gain of channel function. The three variants identified in ID patients without seizures, p.R937C, p.L611Vfs*35 and p.W1716*, did not produce measurable currents. Homology modeling of the missense variants predicted structural impairments consistent with the electrophysiological findings.

**Conclusions:**

Our findings support the hypothesis that complete loss-of-function variants lead to ID without seizures, small gain-of-function variants cause BFNIE and EE variants exhibit variable but profound Na_v_1.2 gating changes. Moreover, structural modeling was able to predict the severity of the variant impact, supporting a potential role of structural modeling as a prognostic tool. Our study on the functional consequences of *SCN2A* variants causing the distinct phenotypes of EE, BFNIE and ID contributes to the elucidation of mechanisms underlying the broad phenotypic variability reported for *SCN2A* variants.

**Electronic supplementary material:**

The online version of this article (10.1186/s10020-019-0073-6) contains supplementary material, which is available to authorized users.

## Background

*SCN2A* (OMIM 182390) encodes the pore-forming α-subunit of the voltage gated sodium channel type 2 (Na_v_1.2). It is predominantly expressed at the axon initial segment and nodes of Ranvier of excitatory neurons of the central nervous system and plays a crucial role for axon potential initiation and propagation in early development (Kaplan et al. 2001, Kole and Stuart 2012). At age 1–2 years, it is partially replaced by Na_v_1.6 (encoded by *SCN8A*) and its main function is then thought to be related to the support of backpropagating action potentials (Hu et al. 2009, Sanders et al. 2018). Variants in *SCN2A* were initially identified as disease cause in patients with generalized epilepsy with febrile seizures plus (GEFS+) (Sugawara et al. 2001) and benign familial neonatal-infantile epilepsy (BFNIE) (Heron et al. 2002). In recent years, *SCN2A* has been recognized as one of the most frequently mutated genes in neurodevelopmental disorders including autism spectrum disorders (ASD), intellectual disability (ID), developmental and epileptic encephalopathy (DEE) and schizophrenia (Li et al. 2016).

Given the broad clinical spectrum associated with pathogenic variants in this gene, understanding the genotype-phenotype correlation is a prerequisite for proper treatment and genetic counselling. To date, categorization of *SCN2A* variants by variant type and location allows limited prediction of the associated phenotype. Causative variants observed in BFNIE and DEE are usually missense variants clustering in the transmembrane segments and short connecting loops (Ben-Shalom et al. 2017), while more than half of the ASD-associated *SCN2A* variants are predicted to introduce a premature stop codon (Ben-Shalom et al. 2017). However, there are also reports of patients with nonsense, frameshift and splice site variants showing a variety of seizure types (Wolff et al. 2017). Electrophysiological analyses of about 20 pathogenic variants mostly in HEK cells and computational modeling of neuronal excitability suggest a gain of channel function and modest neuronal hyperexcitability in BFNIE, while DEE pathogenic variants show more marked and variable gating changes with pronounced hyper- or hypoexcitability predicted by in-silico modeling of six variants (Sugawara et al. 2001, Kamiya et al. 2004, Scalmani et al. 2006, Xu et al. 2007, Misra et al. 2008, Ogiwara et al. 2009, Liao, Anttonen, et al. 2010a, Liao, Deprez, et al. 2010b, Lossin et al. 2012, Lauxmann et al. 2013, Schwarz et al. 2016, Wolff et al. 2017, Berecki et al. 2018, Lauxmann et al. 2018). Gain of function variants were significantly associated with seizure onset before 3 months of age and a better response to sodium channel blockers, while loss of function variants were more often observed with later onset epilepsy and no or even adverse responses to sodium channel blockers (Wolff et al. 2017). One study investigating *SCN2A* variants associated with ASD (with or without seizures) in HEK cells showed partial or complete loss of channel function in all variants (Ben-Shalom et al. 2017). A recently proposed bidirectional hypothesis suggesting that gain of channel function leads to epileptic phenotypes whereas loss of channel function results in ID/ASD phenotypes (Ben-Shalom et al. 2017, Sanders et al. 2018) has been challenged by conflicting findings indicating hypoexcitability in infantile epileptic encephalopathy (EE) (Berecki et al. 2018). Therefore, characterization of additional *SCN2A* variants should help to improve genotype-phenotype correlation eventually enabling mechanism-based therapy (Berecki et al. 2018, Sanders et al. 2018, Ben-Shalom et al. 2017, Wolff et al. 2017).

Here, we investigated the functional consequences of six *SCN2A* variants identified in patients with ID without seizures, early-onset EE or BFNIE using whole-cell patch-clamp experiments in HEK293T cells transiently expressing wild-type or mutant Na_v_1.2. Furthermore, the description of homologous structures in 2017 (Yan et al. 2017, Shen et al. 2017) allowed us to generate a 3D-model for SCN2A and assess the impact of the different variants on protein structure. We further re-evaluated the first family reported with a pathogenic *SCN2A* variant causing DEE (p.R102*) (Kamiya et al. 2004) which stands out as the only functionally studied truncating variant leading to DEE.

## Methods

### Patients and variants

Five previously reported patients carrying a pathogenic *SCN2A* variant (Hackenberg et al. 2014, Rauch et al. 2012, Wolff et al. 2017, Papuc et al. 2018) with a phenotype of either early-onset EE (*n* = 2) or ID without seizures (*n* = 3) were included in this study for functional and clinical evaluation. One BFNIE variant previously studied extensively by others was also investigated as a reference.

### Whole exome sequencing

To re-evaluate the DEE case published by Kamiya et al. in 2004 carrying the p.R102* variant detected by targeted sequencing of *SCN2A*, we now performed trio whole exome sequencing (WES) of this family (see Additional file 1).

### Mutagenesis and cell culture

Human *SCN2A* (‘adult’ isoform (Kasai et al. 2001), RefSeq NM_021007 (O’Leary et al. 2016)), *SCN1B* (NM_001037) and *SCN2B* (NM_004588) cDNA in pCMV6-XL4/5 expression vectors were purchased from OriGene (Rockville, MD, USA). Mutagenesis was performed by Creative Biolabs (Shirley, NY, USA) and verified by sequencing. HEK293T cells (ATCC-LGC Standards, Manassas, VA, USA) were grown in DMEM supplemented with 9% fetal bovine serum and 1% Pen Strep (all Gibco, Carlsbad, CA, USA). Cells were transiently transfected using Lipofectamine LTX with PLUS Reagent (Invitrogen, Carlsbad, CA, USA). Wild-type or mutated Na_v_1.2 α-subunit (1 μg) was co-expressed with β1- and β2-subunits and EGFP (in a ratio of 1:1:1:0.5, respectively). Recordings were made exclusively from EGFP-positive cells.

### Electrophysiology

Whole-cell voltage-clamp recordings were performed at room temperature 18–30 h after transfection using 2–4 MΩ borosilicate glass pipettes, an EPC7 amplifier and Patchmaster v2.11 software (HEKA Elektronik Dr. Schulze GmbH, Lambrecht, Germany). The pipette solution contained (in mM) 110 CsF, 10 NaF, 20 CsCl, 2 EGTA and 10 HEPES ~ 310 mOsm (pH 7.35 with CsOH). Bath solution consisted of (in mM) 145 NaCl, 4 KCl, 1.8 CaCl_2_, 1 MgCl_2_ and 10 HEPES ~ 310 mOsm (pH 7.35 with NaOH). Cells with peak current amplitudes < 0.6 nA were not used for analysis of biophysical parameters due to possible contamination with endogenous currents of HEK cells. Whole-cell capacitance was determined by integrating the area under the capacitive currents in response to a hyperpolarizing voltage step*.* Capacitive transients were compensated electronically. Leak currents were subtracted by use of a P/N procedure. Sodium currents were evoked by 10 ms voltage steps from − 100 mV to + 50 mV from a holding potential of − 120 mV. Current densities were obtained by dividing the peak currents by the capacitance. Charge transfer was calculated as the area under the current trace and normalized to the capacitance. Sodium conductance (G_Na_) was calculated as G_Na_ = I_Na_ / (V_m_ − V_rev_), where I_Na_ is the measured peak current at the test potential V_m_, and V_rev_ is the calculated sodium reversal potential. To obtain activation curves (conductance-voltage relationship), the normalized conductance was plotted against the test potentials and fitted with a Boltzmann sigmoidal function for quantitative analysis. The 10–90% rise time was obtained from the I-V current traces. Fast and slow inactivation time constants were obtained by fitting a second order exponential function to the current decay of the I-V current traces. Steady-state inactivation was determined using 500 ms conditioning prepulses to potentials varying from − 130 to + 10 mV followed by a depolarizing test pulse to + 5 mV. For quantitative analysis, normalized peak currents were plotted against prepulse potential and fitted with a Boltzmann sigmoidal function. Recovery from fast inactivation was recorded by a two-pulse protocol from a holding potential of − 90 mV to + 5 mV with increasing interpulse intervals. For quantitative analyses, the curves were fitted with a first order exponential function where the tau represents the recovery time constant. To analyze use-dependent inactivation, depolarizing pulse trains from − 90 mV to + 10 mV at different frequencies were applied and the residual peak current of the last pulse was normalized to the peak current of the first pulse.

Results are presented as mean ± SEM. Statistical significance of differences in reference to wild-type was calculated by using the unpaired Student’s t test and a significance level of *p* < 0.05. Data were analyzed using NeuroMatic v2.00 (Rothman and Silver 2018) within Igor Pro 6.3 software (WaveMetrics, Portland, OR, USA) and GraphPad Prism 7.0 software (GraphPad Software, La Jolla, CA, USA).

### Protein modeling

The structure of SCN2A was modeled with Modeller 9.16 (Webb and Sali 2017) using the recently published structure of a homologous voltage-gated sodium channel (PDB: 5X0M (Shen et al. 2017)) as template. Residues 440–741 and 990–1184 were excluded from the modeling procedure because they were either absent in the template or they did not exhibit a sufficient sequence similarity between template and target sequence. RasMol (Sayle and Milner-White 1995) was used for structure analysis and visualization.

## Results

### Patient phenotypes and variants

**Fig. 1:**
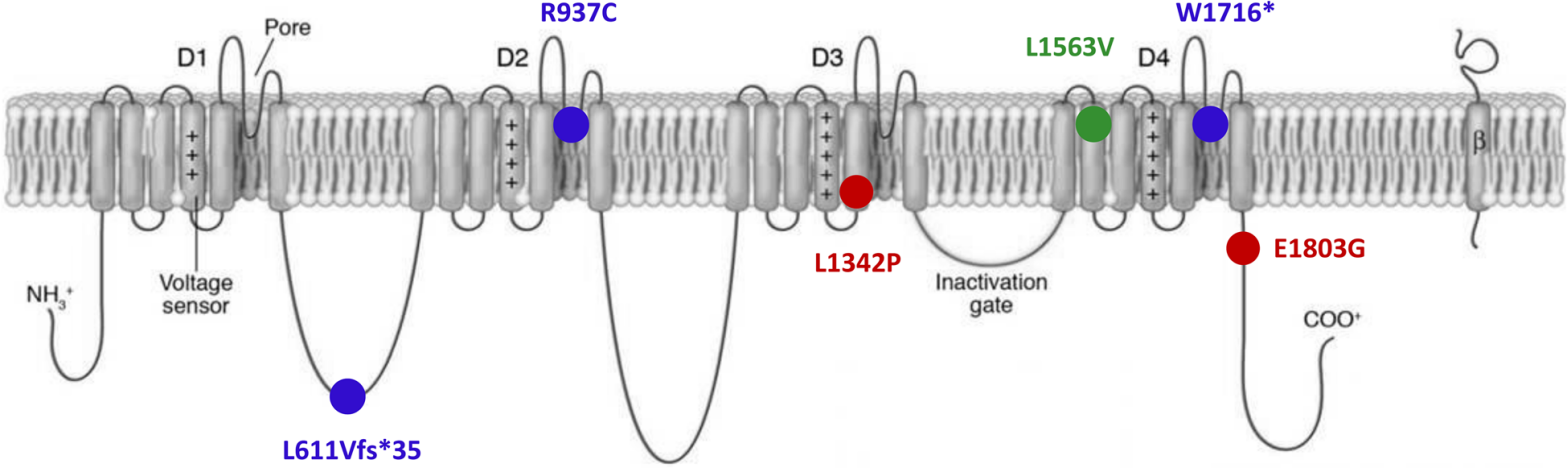
Schematic drawing of SCN2A indicating the location of studied pathogenic variants. The three patients with ID without seizures carried de novo heterozygous variants in *SCN2A* (shown in blue), two of which are nonsense variants (p.L611Vfs*35 and p.W1716*) and one is a missense variant in the pore-forming loop of domain 2 of the channel (p.R937C). The two patients with early onset EE harbored de novo heterozygous missense variants (shown in red) in the fifth transmembrane segment of the third channel domain (p.L1342P) or the C-terminal domain (p.E1803G), respectively. The heterozygous inherited missense variant previously reported by others in a family with BFNIE (Heron et al. 2002) (shown in green) is located in the second transmembrane segment of domain 4 (p.L1563V). Adapted from Meisler and Kearney (2005)

Comprehensive phenotypes of our patients and bioinformatic annotations of their respective variants are presented in Table 1 and their location in the 2D-scheme of the Na_v_1.2 channel is depicted in Fig. 1. All missense variants affect a highly conserved amino acid and have damaging in-silico predictions.

**Table 1:** Molecular data and patient phenotypes

	Patient 1 / ID	Patient 2 / ID	Patient 3 / EE	Patient 4 / ID	Patient 5 / EE	Published BFNIE family
Previous publication	Rauch et al. 2012 ER8490	Rauch et al. 2012 ZH60991	Hackenberg et al. 2014 ZH62611	Wolff et al. 2017 Patient 63	Papuc et al. 2018 ID42680	Heron et al. 2002; Functional characterization by Scalmani et al. 2006, Xu et al. 2007, Misra et al. 2008, Berecki et al. 2018
Additional reports on the same variant	Li et al. 2016 (due to a repeat shift described as p.D609fs), ID	Functional studies by Ben-Shalom et al. 2017	Dimassi et al. 2016 Matalon et al. 2014 Wolff et al. 2017 All EE	–	–	
Type of variant	het. frameshift	het. missense	het. missense	het. stop	het. missense	het. missense
Inheritance	de novo	de novo	de novo	de novo	de novo	Inherited from a parent
cDNA level^a^	c.1831_1832delCT	c.2809C>T	c.4025T>C	c.5147G>A	c.5408A>G	c.4687C>G
Protein level	p.(L611Vfs*35)	p.(R937C)	p.(L1342P)	p.(W1716*)	p.(E1803G)	p.(L1563V)
rs number (dbSNP^b^)	unreported	rs796053197	rs796053134	unreported	unreported	rs121917750
Chromosomal position (hg19)	chr2:166179821–166179822	chr2:166201311	chr2:166231247	chr2:166245463	chr2:166245724	chr2:166243391
Allele Frequency (GnomAD^c^)	unreported	unreported	unreported	unreported	unreported	unreported
Conservation^d^, location in protein	/	high, DII S5–6	high, DIII S5	/	high, C-terminus	high, DIV S2
Established Prediction tools^e^	/	probably damaging	probably damaging	/	probably damaging	probably damaging
Age at last investigation	18y 1m	22y 9m	6y 8m	24y 4m	16y 4m	
Gender	male	female	female	female	male	
Consanguinity, ethnicity	no, German	no, Swiss	no, Japanese/Swiss	no, Swiss	no, Swiss	
Pre- or perinatal anomalies	none	none	none	none	none	
Gestational age (weeks)	41	40 3/7	38 5/7	term	41 1/7	
Birth weight / length / HC	3600 g / 52 cm / 35 cm	3800 g / 51 cm / NA	2970 g / 48 cm / 36 cm	3150 g / NA / NA	4120 g / 51 cm / NA	
Intellectual disability	yes (IQ 58)	yes (IQ < 50)	yes, severe	yes, mild reading capital letters, calculating with numbers up to 10	yes, severe	
Autistic features	yes	yes	NA	yes	NA	
Other abnormal behavior	compulsive stereotypies, autoaggressive behavior	compulsive stereotypies, autoaggressive behavior	none	recurrent episodes of mutism and psychotic episodes	none	
Developmental delay Age of indep. walking Age of first words Speech development	yes 13 m 12 m Speaks sentences	yes 24 m 24 m Simple sentences	yes no indep. sitting or walking - no speech	yes 24 m delayed	yes no indep. sitting or walking - no speech	
Seizures (onset)	none	none	yes (diagnosis at 5 m)	none	yes (day 3)	
Seizure type	/	/	myoclonic / tonic	/	myoclonic / tonic	
Course of epilepsy	/	/	at least 3 seizures per day, pharmacoresistant	/	1–7 seizures per day, pharmacoresistant	
Acquired microcephaly	no	no	yes	no	no	
Muscle tone abnormalities, movement disorders	none	hypotonia	severe hypotonia, choreoathetotic movements	none	spasticity, bilateral contractures of knees	
Dysmorphisms	long face, narrow palpebral fissures, and relatively thick eyebrows, narrow palate, inverted nipples, tapering fingers	long face, narrow palpebral fissures, mild brachymetacarpalia Dig. IV-V, tapering fingers	none	NA	high, narrow palate	
Other health problems	hyperopia	myopia	OSAS, scoliosis, hip dysplasia left, hip subluxation right, recurring respiratory infections	none reported	cortical visual impairment, gastro-jejunal tube	
EEG	normal	NA	multifocal sharp wave activity, hypsarrhythmia	normal	frontocentral spike-wave activity on both sides, burst suppression	
MRI	pinealis cyst	NA	progressive brain atrophy	normal	normal at age 4d, generalized supra- and infratentorial atrophy, bilateral hippocampal atrophy, left-sided hippocampal sclerosis and atrophy of corpus callosum at age 14y	

Abb: / not relevant for this case; *D* domain, *het* heterozygous, *EEG* electroencephalogram, *HC* head circumference, *m* months, *MRI* magnetic resonance imaging, *NA* not available, *S* segment; *y* years

^a^according to reference sequence NM_021007

^b^ https://www.ncbi.nlm.nih.gov/SNP

^c^ http://gnomad.broadinstitute.org

^d^according to Alamut Visual v.2.8.1

^e^Polyphen2, MutationTaster, SIFT assessed by Alamut Visual v.2.8.1

### ID mutants are non-conducting

**Fig. 2:**
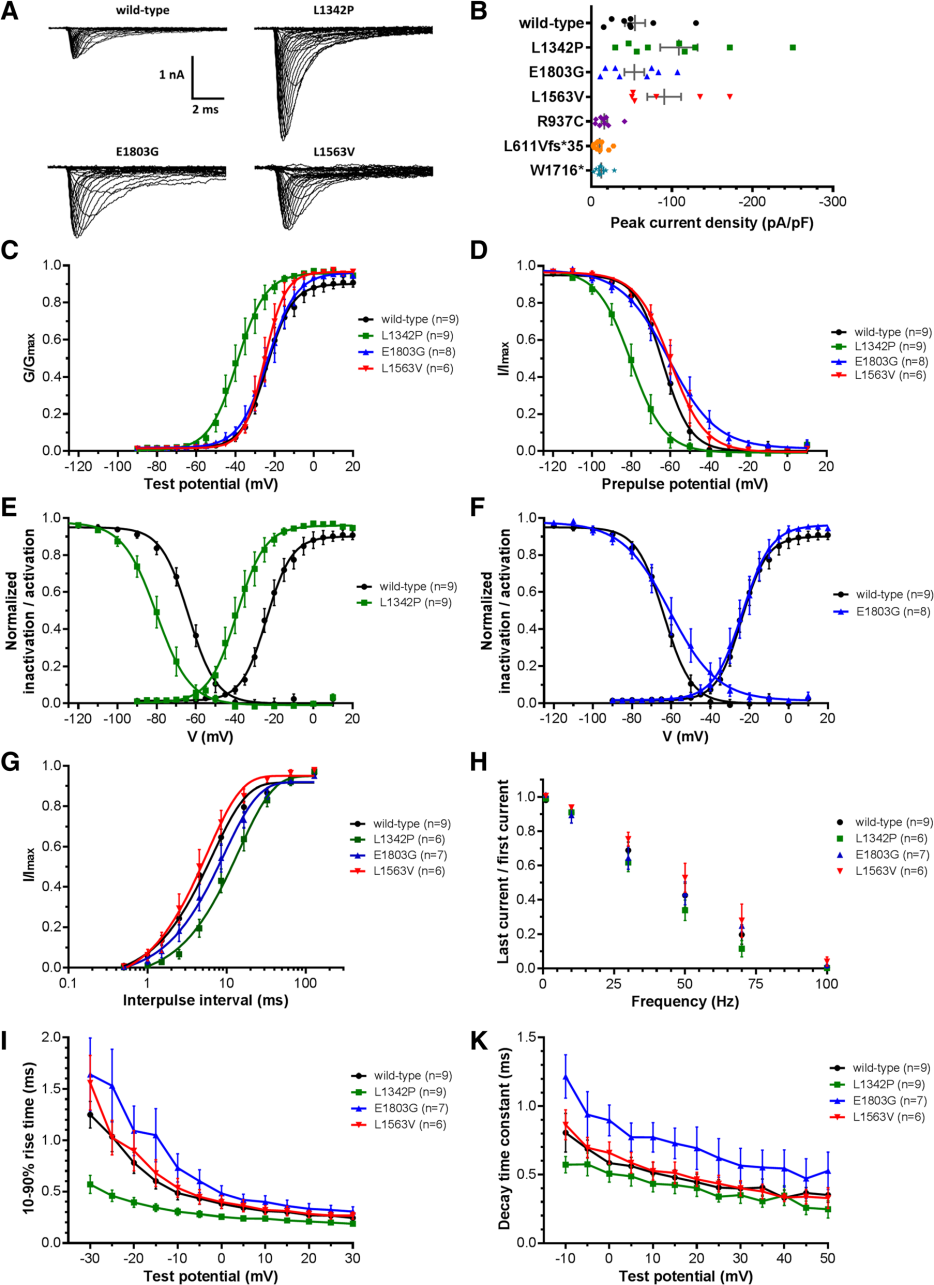
Patch-clamp recordings in HEK293T cells expressing recombinant Na_v_1.2 wild-type or mutant channels. Variants E1803G (blue) and L1342P (green) have been detected in patients with EE, L1563V (red) in a family with BFNIE, and R937C (purple), L611Vfs*35 (orange) and W1716* (light blue) cause ID. **a** Family of whole-cell sodium currents of wild-type and conducting mutant Na_v_1.2. **b** Peak current amplitudes normalized to cell capacitance (bars are mean ± SEM) were not significantly different. **c** Voltage dependence of activation obtained by plotting the normalized conductance against test potentials and fitted with a Boltzmann function. L1342P showed a significant negative shift (*p* < 0.001). **d** Voltage dependence of steady-state inactivation obtained by displaying the normalized current amplitude against the prepulse potential fitted with a Boltzmann function. A negative shift was observed for both L1342P (*p* < 0.001) and L1563 V (*p* = 0.017), while E1803G had a shallower slope (*p* < 0.001). **e** Activation and inactivation curves of L1342P in comparison to wild-type demonstrating a hyperpolarized shift with unchanged activation-inactivation coupling. **f** Activation and inactivation curves of E1803G in comparison to wild-type illustrating the increase of the window current. **g** Time-dependent recovery from inactivation fitted with a single exponential function and plotted against a logarithmic time scale. A significantly slower recovery rate was observed in L1342P (*p* = 0.002). **h** Use-dependent inactivation. The frequencies of depolarizing pulse trains are plotted against the residual peak current amplitude of the last pulse normalized to the peak current amplitude of the first pulse. **i** 10–90% rise time depicted against test potentials. L1342P exhibited a significantly faster rise time at the voltage step to − 30 mV (*p* = 0.02). **k** Fast inactivation time constants plotted against test potentials

Representative families of whole-cell current traces of wild-type and conducting mutant channels are shown in Fig. 2a, while Fig. 2b depicts the peak current amplitude normalized by cell capacitance for wild-type and mutant Na_v_1.2. Recombinant Na_v_1.2 channels carrying variants from ID patients showed no or only small residual currents indistinguishable from endogenous HEK293T cell currents (Zhu et al. 1998, Cummins et al. 1993) (mean peak current wild-type = − 1131 ± 190 pA, *n* = 8; R937C = − 271 ± 46 pA, *n* = 9; L611Vfs*35 = − 258 ± 52 pA, *n* = 10; W1716* = − 253 ± 45 pA, *n* = 8). Thus, ID variants resulted in a complete loss of Na_v_1.2-mediated current. The mean peak current density of the EE and BFNIE Na_v_1.2 mutants did not differ significantly from the wild-type (Fig. 2b, Table 2). Striking alterations were observed in other functional characteristics as described below.

**Table 2:** Biophysical parameters of Na_v_1.2 wild-type and mutants

	Peak current density		Voltage dep. of steady-state inactivation			Voltage dep. of steady-state activation			Recovery from inactivation		10–90% rise time		Fast decay time constant	
	Mean peak amplitude (pA/pF)	n	V_1/2_ (mV)	*k*	n	V_1/2_ (mV)	*k*	n	τ_rec_ (ms)	n	t at 0 mV (ms)	n	τ_fast_ at 0 mV (ms)	n
wild-type	−54.24 ± 12.60	8	−63.53 ± 0.79	−6.91 ± 0.69	9	−23.87 ± 0.64	6.46 ± 0.57	9	7.02 ± 0.86	9	0.38 ± 0.05	9	0.58 ± 0.07	9
L1342P	− 108.80 ± 23.20	9	−79.71 ± 1.18**	− 8.30 ± 1.02	9	−39.07 ± 0.82**	7.25 ± 0.73	9	15.03 ± 2.18*	6	0.26 ± 0.03	9	0.51 ± 0.06	9
E1803G	−53.48 ± 12.28	8	−60.90 ± 1.87	−12.35 ± 1.00**	8	−23.61 ± 0.88	7.38 ± 0.78	8	10.04 ± 1.91	7	0.48 ± 0.08	7	0.90 ± 0.11	7
L1563V	−90.55 ± 20.97	6	−59.77 ± 1.21*	−8.75 ± 1.07	6	−25.17 ± 0.61	5.37 ± 0.53	6	6.05 ± 1.27	6	0.40 ± 0.06	6	0.66 ± 0.08	6

Values significantly different from wild-type are indicated as follows ∗*p* < 0.05, ∗∗*p* < 0.001

### The EE mutant L1342P shows a hyperpolarizing shift of activation and inactivation curves

Voltage-dependent activation and inactivation curves of L1342P exhibited a hyperpolarizing shift of about 15 mV relative to wild-type (Fig. 2c-e, Table 2). Furthermore, this mutant showed a slower recovery time constant from inactivation (Fig. 2g, Table 2) and a trend towards faster rise time kinetics, although reaching significance only at − 30 mV (Fig. 2i, Additional file 1: Table S1). Differences in the current density, decay kinetics and use-dependent inactivation were not statistically significant (Fig. 2b, h and k, Table 2, Additional file 1: Table S2). Therefore, this mutant showed mixed gain and loss of function effects.

### The EE mutant E1803G exhibits a shallower slope of the inactivation curve that leads to an increased window current

While no changes in voltage dependence of activation were observed for the mutant E1803G compared to wild-type, the slope of the voltage dependence of inactivation was significantly less steep (Fig. 2c-d, Table 2), leading to a larger overlap of activation and inactivation curves. This increased window current suggests the presence of a persistent current at membrane voltages between − 45 to − 25 mV (Fig. 2f). Additionally, the E1803G mutant tended to have slower activation and inactivation kinetics (Fig. 2i-k, Additional file 1: Table S1) and hence tended to let more charge pass compared to wild-type channels (mean charge transfer normalized to conductance at a test potential of − 10 mV: wild-type = 0.09 ± 0.02 C/F, *n* = 8; E1803G = 0.16 ± 0.03 C/F, *n* = 8; *p* = 0.092). The use-dependent inactivation was similar to wild-type (Fig. 2h, Additional file 1: Table S2). The larger window current may increase neuronal excitability and thereby promote the presence of epileptic seizures in affected patients.

### The BFNIE mutant L1563V exhibits a small depolarizing shift of the inactivation curves

Activation, recovery from inactivation, kinetics and use-dependent inactivation did not show relevant differences between L1563V and wild-type channels (Fig. 2, Table 2 and Additional file 1: Tables S1–2). However, the inactivation curve was slightly but significantly shifted by about 4 mV in the depolarizing direction (Fig. 2d, Table 2), which suggests a small gain of channel function.

### Structural modeling of the ID variant R937C predicts a destabilizing effect on the selectivity filter

**Fig. 3:**
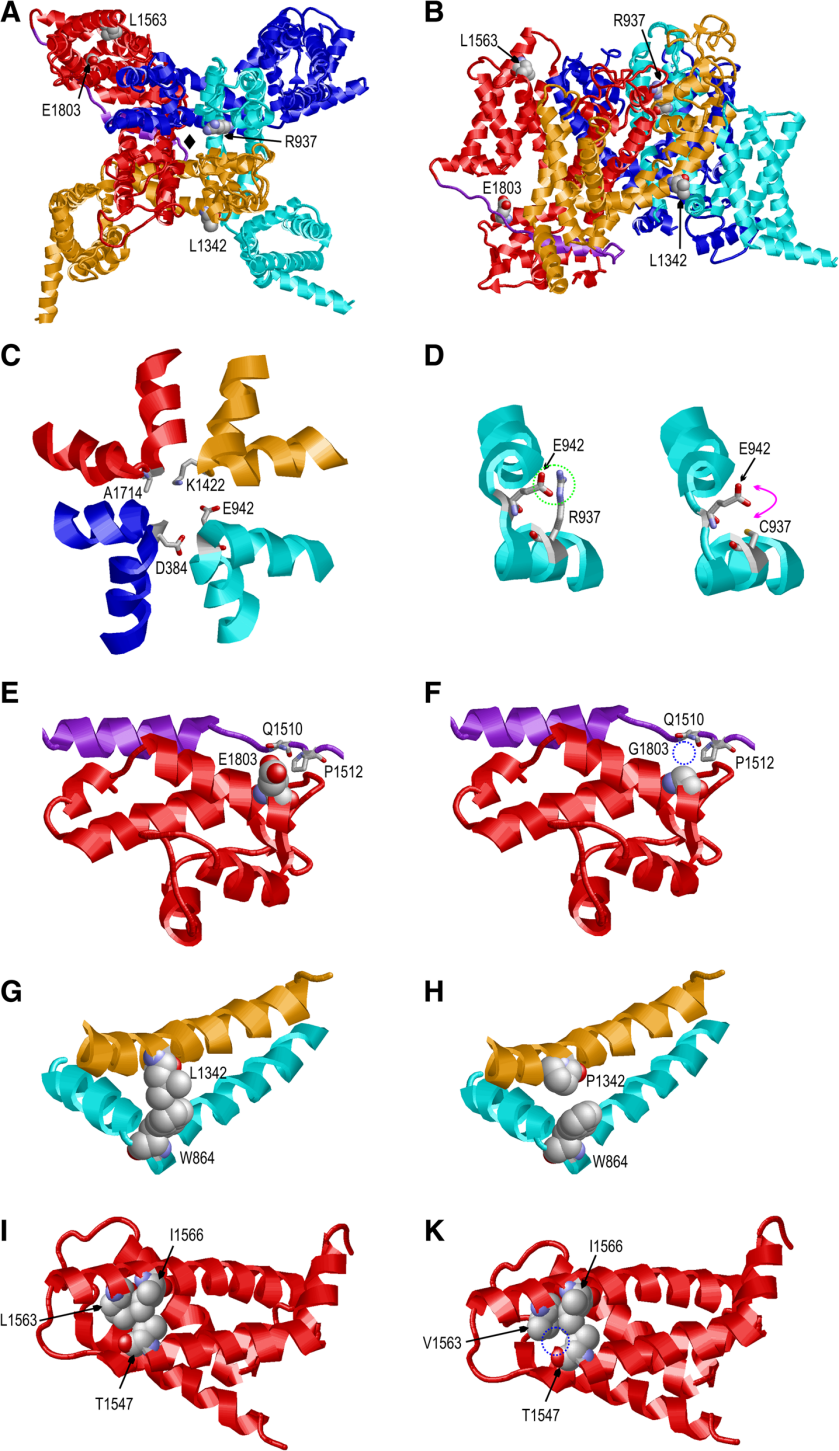
Effect of amino acid exchanges on the SCN2A structure. **a** Top view on a model of the SCN2A structure. The four homologous repeats are shown in different colors (blue, cyan, orange, red) and the III-IV domain linker is shown in purple. The site of the pore is marked by a black diamond and the positions of the missense variants investigated in the present study are indicated. The extracellular sequence stretch spanning residues 275–359 has been omitted for clarity. **b** Side-view on the SCN2A structure. Color coding as in (**a**). **c** Model of the SCN2A pore region. The four residues that are critical for Na^+^ selectivity are shown in stick presentation. **d** In the wild-type SCN2A (left panel) the sidechain orientation of E942, which is part of the selectivity filter, is stabilized by polar interactions with R937 (see encircled region). These interactions cannot be formed in the R937C variant (right panel) by the shorter and uncharged cysteine sidechain thus impeding fixation of the E942 sidechain (flexibility is indicated by the magenta arrow). **e** E1803 forms interactions with residues Q1510 and P1512 of the III-IV domain linker, which cannot be formed in the (**f**) E1803G variant. The lacking interactions are highlighted by a blue dotted circle. **g** L1342 forms sidechain interactions with W864 of the adjacent repeat. **h** In the L1342P variant, these interactions cannot be formed by the less-extended proline sidechain. **i** L1563 forms hydrophobic interactions within the fourth domain. **k** These interactions are partially lost in the L1563V variant by the shorter valine sidechain (indicated by a blue dotted circle)

The α-subunits of voltage-gated sodium channels contain four homologous repeats, which contribute to the formation of one central pore region (Fig. 3a-b). The SCN2A pore region contains the typical DEKA motif, which is formed by four conserved Asp/Glu/Lys/Ala residues (Fig. 3c). These residues represent the sodium selectivity filter and are therefore crucial for channel function (Shen et al. 2017). The position of one of these residues (E942) is stabilized by electrostatic interactions with the adjacent R937 (Fig. 3d). In the R937C variant these interactions cannot be formed by the shorter and uncharged cysteine sidechain resulting in a less defined position of the E942 sidechain. These structural changes offer an explanation for the electrophysiologically observed loss of conductance.

### Structural modeling of the EE variant E1803G supports altered inactivation properties

Residue E1803 is located in the globular C-terminal domain of SCN2A and forms sidechain interactions with two residues of the III-IV domain linker, which cannot be formed in the E1803G variant (Fig. 3e-f). The III-IV linker plays a key role for fast inactivation of voltage-gated sodium channels (Shen et al. 2017, Yan et al. 2017) suggesting that an altered interaction with adjacent domains, as observed for the E1803G variant, might affect inactivation properties.

### The EE variant L1342P is predicted to disturb interactions with the adjacent S4-S5 linker

Residue L1342 is located in helix S4 and the effect of a L1342P exchange has been assessed previously based on the isolated helix (Hackenberg et al. 2014) due to the lack of a homologous 3D-structure at that time. This previous study revealed the lack of a backbone hydrogen bond in the mutant resulting in helix destabilization (Hackenberg et al. 2014). The availability of homologous structures in 2017 (Yan et al. 2017, Shen et al. 2017) allowed the generation of a complete 3D-model for SCN2A and a more comprehensive evaluation of the effects of the L1342P variant. An inspection of the SCN2A model reveals that L1342 forms sidechain interactions with W864, which is located in the S4-S5 linker of the adjacent repeat (Fig. 3g). These interactions cannot be formed in the L1342P variant because the cyclic proline sidechain cannot adopt an extended conformation required for W864 interaction (Fig. 3h). Based on this significant structural disturbance and the observation that a number of disease-related variants of Na_v_ channels mapped to the interface between S4 and S5 segments in the neighboring repeats (Shen et al. 2017, Huang et al. 2017), L1342P is also likely to affect the biophysical properties of SCN2A.

### Hydrophobic interactions within the fourth domain are expected to be diminished in the L1563V BFNIE variant

Residue L1563 is located in helix S2 of the fourth voltage-sensing domain and forms hydrophobic sidechain interactions in the core of the domain (Fig. 3i). In the L1563V variant some of these hydrophobic interactions are lost due to the shorter valine sidechain, but the overall packing of helices S1 and S2 remains unaffected (Fig. 3k). Thus, this amino acid change is expected to have only a moderate effect on the local structure of the fourth domain.

### Trio WES in the published DEE patient carrying the R102* variant did not reveal a second hit

The p.R102* variant is to date the only functionally evaluated protein truncating *SCN2A* variant that leads to an encephalopathic phenotype with seizures (Kamiya et al. 2004). The reported patch-clamp studies showed a complete loss of channel function for this variant. This patient was reported to exhibit intractable seizures with an onset at age 1y 7m, ASD and severe ID. However, the mother was also reported to have suffered from recurrent febrile seizures until the age of 6y, suggesting the possibility of a second, maternally inherited variant in an epilepsy gene to cause the seizures in the patient and her mother while the de novo truncating *SCN2A* variant could explain the ASD/ID phenotype. This hypothesis is further supported by a recent report of a second patient carrying this variant (Monies et al. 2017) with a phenotype of severe ID and ASD but no seizures at the age of 6.5 years (personal communication Fowzan S. Alkuraya). To re-evaluate this case, we performed WES in the patient and her parents and filtered for dominant maternally inherited and recessive rare variants in known epilepsy and ID genes, as well as de novo variants. However, we did not detect a likely pathogenic sequence or copy number variant in any known epilepsy gene other than the already known *SCN2A* de novo variant.

## Discussion

All three variants from patients with ID without seizures studied here showed a complete loss of Na_v_1.2-mediated currents. While the truncating variant L611Vfs*35 is likely to result in nonsense mediated mRNA decay (NMD) in vivo, the stop variant W1716* is located in the last exon and the mRNA should therefore not be subject to NMD (Sanders et al. 2018), but is expected to direct the synthesis of a C-terminally truncated protein. Thus, the complete loss of function in this mutant must be due to other mechanisms such as protein instability or disturbed transport. In line with this concept, Ben-Shalom et al. (2017) showed that in all three investigated truncating *SCN2A* variants no channel could be detected in the membrane of overexpressing HEK293 cells by immunocytochemistry. Of note, multiple variants truncating the protein at later positions (E1777, K1863, I1877) have also been reported to result in complete loss of conductance (Mantegazza et al. 2001).

It was less apparent how the missense variant R937C caused a complete loss of function, as the R937C mutant protein has been previously shown to be correctly located in the cell membrane (Ben-Shalom et al. 2017). Its critical location in the pore loop let us speculate that the pore could be completely blocked by the amino acid substitution. Our homology model supports this idea, predicting that R937 stabilizes the glutamate residue which is an essential part of the selectivity filter of Na_v_1.2. The same structural mechanism also applies to the previously described R937H variant causing ASD, which also exhibited no sodium flux (Ben-Shalom et al. 2017). Interestingly, two other variants for which no or reduced sodium conductance was reported (Ben-Shalom et al. 2017) are also located in the immediate vicinity of the DEKA selectivity filter (Fig. 3c). R379H is located near D384 and T1420M near K1422 of the selectivity filter. Thus, it is likely that these variants interfere with sodium permeability by a mechanism similar to that deduced above for the R937C variant.

To date, only one other study has functionally evaluated *SCN2A* variants with a non-epileptic phenotype (Additional file 1: Table S3) (Ben-Shalom et al. 2017). In their analysis of variants associated with ASD, all truncating variants and most missense variants were non-conducting, including R937C also investigated in our study. Notably, all non-conducting variants were detected in patients without additional seizures.

Both variants causing a phenotype of EE led to profound changes of Na_v_1.2 channel function. While L1342P affected several gating properties, E1803G mainly showed an increased window current caused by a shallower slope of the inactivation curve. So far, 10 *SCN2A* variants detected in EE patients have been studied electrophysiologically (Additional file 1: Table S3) (Kamiya et al. 2004, Ogiwara et al. 2009, Liao, Anttonen, et al. 2010, Lossin et al. 2012, Wolff et al. 2017, Berecki et al. 2018, Lauxmann et al. 2018). Two of them, E1211K and R1312T, exhibited gating changes similar to the ones observed in our mutant L1342P with a negative shift in both activation and inactivation curves and slowed recovery from inactivation (Ogiwara et al. 2009, Lossin et al. 2012). Interpretation based on these profoundly altered biophysical properties alone is challenging. However, a computational model of a cortical pyramidal neuron carrying the E1211K variant showed a marked neuronal hyperexcitability with a hyperpolarized spike threshold and an increased spike rate (Additional file 1: Table S4) (Ben-Shalom et al. 2017). We therefore also assume an increase in excitability for neurons carrying the L1342P variant. To date, three other patients carrying the de novo variant L1342P have been reported (Matalon et al. 2014, Dimassi et al. 2016, Wolff et al. 2017). All four affected patients share a common phenotype with an early seizure onset between age 3 to 6 months, severe ID, muscle tone abnormalities, (progressive) brain atrophy in MRI, and no eye contact (3/4) or cortical visual impairment (1/4). The seizures were intractable in 3/4 patients, and the EEG showed hypsarrhythmia in all three patients where EEG was reported and multifocal epileptic activity in 2/3. Acquired microcephaly and choreiform movements were noted in 2/4 patients.

Several missense variants located in the cytoplasmic C-terminal domain (CTD) leading to EE have been reported (Ben-Shalom et al. 2017). Interestingly, the only functionally studied variant, R1882Q, was found to have altered activation and inactivation curves leading to an increased window current, similar to our findings for E1803G. Our structural model predicts that the contacts between the CTD and the inactivation gate are disturbed (Fig. 3e-f), providing a mechanistic explanation for the altered inactivation properties observed in the E1803G mutant. Interestingly, gating changes leading to a persistent current seem to be a recurrent pathomechanism for *SCN2A* variants detected in EE patients, as this dysfunction has also been described previously as the mechanism of disease for the EE variants A263V (Liao, Anttonen, et al. 2010a) and V423L (Wolff et al. 2017). This pathomechanism was also observed in hippocampal pyramidal neurons of a Q54 transgenic mouse model carrying a gain-of-function *Scn2a* variant and showing a progressive epilepsy phenotype (Kearney et al. 2001). Persistent currents have been found to substantially influence action potential thresholds in axons and are considered to be important for generation of high-frequency action potential bursts in axons and nodes of Ranvier, the main expression sites of Na_v_1.2 (Kole and Stuart 2012).

Most previous electrophysiological studies of BFNIE variants reported a gain-of-function effect, assuming hyperexcitability as the mechanism of disease (Additional file 1: Table S3) (Scalmani et al. 2006, Xu et al. 2007, Misra et al. 2008, Liao, Deprez, et al. 2010b, Lauxmann et al. 2013, Lauxmann et al. 2018, Berecki et al. 2018). Our results for the recurrent BFNIE variant L1563V of a small but significant depolarizing shift of the inactivation curve expected to result in a small gain-of-function are in line with previous studies of this variant (Scalmani et al. 2006, Xu et al. 2007, Misra et al. 2008, Berecki et al. 2018). Consistent with the small effect size, molecular modeling (Fig. 3i-k) showed that the L1563V exchange causes only a small perturbation of the SCN2A structure. In BFNIE, seizures are thought to be self-limited due to the developmental regulation of sodium channels with Na_v_1.2 being partially substituted by Na_v_1.6 (Liao, Deprez, et al. 2010b, Lauxmann et al. 2013, Sanders et al. 2018), while the more pronounced impact of DEE variants on gating properties also observed in this study suggests continued disturbance of neuronal function (Ben-Shalom et al. 2017).

Our study of six *SCN2A* variants representing the phenotypic variability known for this gene supports the association of their respective functional impact with the observed patient phenotype. Our data corroborates the assumption that 1) a loss of channel function caused by de novo truncating or missense variants is the underlying mechanism that leads to ID 2) benign epilepsy is due to usually inherited missense variants that lead to a small but relevant gain of channel function and 3) epileptic encephalopathies are the result of profound and lasting gating changes due to de novo missense variants (Ben-Shalom et al. 2017, Sanders et al. 2018, Berecki et al. 2018). Additionally, we suggest that a complete loss of Na_v_1.2 conductance is associated with ID/ASD without seizures, as supported by our own data and the results reported by Ben-Shalom et al. (2017), although like in our study long-term follow-up is required since seizures may appear later in childhood. This assumption is in line with data from a heterozygous knock-out mouse model which did not show a seizure phenotype (Planells-Cases et al. 2000, Middleton et al. 2018). However, another study on knock-out mouse models despite the absence of behavioral seizures showed very short absence seizures-like patterns in electrocorticography (Ogiwara et al. 2018), and multiple truncating *SCN2A* variants have been reported in patients with EE that pose exceptions to this proposition (Kamiya et al. 2004, Carvill et al. 2013, Horvath et al. 2016, Moller et al. 2016, Wolff et al. 2017). These truncating variants, though functionally studied in only one case (Kamiya et al. 2004), are expected to result in a complete loss of function and would therefore well explain the developmental delay and mental impairment but not the development of seizures. A closer look at the respective reports revealed that a patient reported with moderate ID and seizures between the age of 4y 7m and 9y by Wolff et al. (2017) and carrying the variant W1716* is the same patient reported in this study, who was suspected but never proven to have seizures of absence type that were later interpreted as rather being mutistic episodes. The patient carrying the de novo variant R102* reported with intractable epilepsy, severe ID and ASD had a mother with recurrent febrile seizures (Kamiya et al. 2004) suggesting an inherited second hit contributing to the seizure phenotype, albeit not detected by our WES analysis. Accordingly, the same variant in an unrelated patient (Monies et al. 2017) with follow-up until age 6.5 years did not cause any seizures (personal communication Fowzan S. Alkuraya). The variant R1435* was reported twice as well, once in a patient with ASD, ID and seizures (Trump et al. 2016) and once in a patient with ID and ASD without seizures at the last follow-up at age 7y 8m (Wolff et al. 2017), also suggesting a possible second hit or other modifier in the first patient. The assumption of a modifying effect of the genetic background is supported by two studies where different strains of mice carrying the same *Scn2a* variant exhibited differences in seizure severity (Kearney et al. 2001, Thompson et al. 2017). In addition, acquired factors such as hypoxia may also impact Na_v_1.2 function (Plant et al. 2016, Winquist and Cohen 2018).

It has recently been shown that voltage-gated sodium channels assemble and gate as dimers (Clatot et al. 2017) and that some pathogenic Na_v_1.5 variants exert a dominant-negative effect through impairing trafficking and gating of the wild-type channel (Clatot et al. 2012, Clatot et al. 2018). A possible dominant-negative effect was also discussed for pathogenic Na_v_1.2 variants since Kamiya et al. (2004) described a reduced whole-cell sodium current when co-expressing the truncating variant R102* together with wild-type. However, this effect disappeared when co-expressing β-subunits. Ben-Shalom et al. (2017) did not find a dominant-negative effect either when testing a non-conducting missense variant. Currently it can only be speculated if some of the variants might exert a dominant-negative effect and thereby could explain some outliers of the assumed genotype-phenotype correlation. Hence, this potential pathomechanism should be kept in mind and addressed in future studies using suitable models resembling the heterozygous constellation in the patient.

Another point to consider regarding the pathomechanism of *SCN2A* variants is the isoform-dependent effect of Na_v_1.2 variants. Next to the ‘adult’ Na_v_1.2 isoform, which was used by all previous electrophysiological investigations utilizing overexpression of human SCN2A as well as in our study, a ‘neonatal’ isoform exists differing only in a single amino acid at position 209 of the protein (Kasai et al. 2001, Gazina et al. 2015). The ‘neonatal’ isoform is expressed at different levels at birth and then decreases until levelling at 10–20% of the ‘adult’ isoform at 20 weeks postnatally in mice (Gazina et al. 2010). Electrophysiological studies indicate that pathogenic variants can exert different effects in this ‘neonatal’ isoform compared to the predominantly studied ‘adult’ isoform (Xu et al. 2007, Liao, Deprez, et al. 2010b).

## Conclusions

In conclusion, our work corroborates a strong genotype-phenotype correlation of deleterious *SCN2A* variants and demonstrates that structural modeling can be a useful tool to predict the severity of the variant impact, suggesting its potential as a useful and fast tool to support variant interpretation in diagnostics. We further illustrate the mechanism of how missense variants located in the pore-forming loop can lead to a loss of channel function and how C-terminally located missense variants can exert a severe disturbance on Na_v_1.2 gating properties.

## Additional file

Additional file 1 Supplementary Methods. **Table S1.** Activation and inactivation kinetics of Na_v_1.2 wild-type and mutants. **Table S2.** Use-dependent inactivation of Na_v_1.2 wild-type and mutants. **Table S3.** Previous electrophysiological studies of disease causing *SCN2A* variants. **Table S4.** Previous studies using in-silico modeling of neuronal excitability of disease causing *SCN2A* variants. (DOCX 46 kb)

## Abbreviations

ASD: Autism spectrum disorders
BFNIE: Benign familial neonatal-infantile epilepsy
CTD: C-terminal domain
DEE: Developmental and epileptic encephalopathy
EE: Epileptic encephalopathy
EEG: Electroencephalography
GEFS+: Generalized epilepsy with febrile seizures plus
ID: Intellectual disability
MRI: Magnetic resonance imaging
Na_v_1.2: Voltage-gated sodium channel type 2
NMD: Nonsense mediated mRNA decay
WES: Whole exome sequencing
