# Supplementary material for: Further corroboration of distinct functional features in SCN2A variants causing intellectual disability or epileptic phenotypes
Source: Mol Med. 2019 Feb 27;25:6. doi: 10.1186/s10020-019-0073-6 (PMC6391808; doi:10.1186/s10020-019-0073-6)
Supplement: Supplementary file 1 — Supplementary Methods. Table S1. Activation and inactivation kinetics of Nav1.2 wild-type and mutants. Table S2. Use-dependent inactivation of Nav1.2 wild-type and mutants. Table S3. Previous electrophysiological studies of disease causing SCN2A variants. Table S4. Previous studies using in-silico modeling of neuronal excitability of disease causing SCN2A variants. (DOCX 46 kb) [file 10020_2019_73_MOESM1_ESM.docx]

**Supplementary material to “Further corroboration of distinct functional features in *SCN2A* variants causing intellectual disability or epileptic phenotypes”**

Anaïs Begemann^1,2^, Mario A. Acuña^2,3^, Markus Zweier^1,2^, Marie Vincent^4^, Katharina Steindl^1,2^, Ruxandra Bachmann-Gagescu^1^, Annette Hackenberg^5^, Lucia Abela^2,5^, Barbara Plecko^2,5,6^, Judith Kroell-Seger^7^, Alessandra Baumer^1^, Kazuhiro Yamakawa^8^, Yushi Inoue^9^, Reza Asadollahi^1,2^, Heinrich Sticht^10^, Hanns Ulrich Zeilhofer^2,3,11,12^†^,^ Anita Rauch^1,2,12,13^†^*^

^1^Institute of Medical Genetics, University of Zurich, 8952 Schlieren-Zurich, Switzerland

^2^radiz—Rare Disease Initiative Zürich, Clinical Research Priority Program for Rare Diseases, University of Zurich, 8006 Zurich, Switzerland

^3^Institute of Pharmacology and Toxicology, University of Zurich, 8057 Zurich, Switzerland

^4^Service de génétique médicale, CHU Nantes, 44093 Nantes, France

^5^Division of Child Neurology, University Children’s Hospital Zurich, 8032 Zurich, Switzerland

^6^Division of General Pediatrics, Department of Pediatrics and Adolescent Medicine, Medical University of Graz, 8036 Graz, Austria

^7^Children’s department, Swiss Epilepsy Centre, Clinic Lengg, 8008 Zurich, Switzerland

^8^Laboratory for Neurogenetics, RIKEN Center for Brain Science, Wako-shi, Saitama 351-0198, Japan

^9^National Epilepsy Center, NHO Shizuoka Institute of Epilepsy and Neurological Disorders, Shizuoka 420-8688, Japan

^10^Institute of Biochemistry, Friedrich-Alexander-Universität Erlangen-Nürnberg (FAU), Erlangen 91054, Germany

^11^Institute of Pharmaceutical Sciences, ETH Zurich, Zürich 8093, Switzerland

^12^Neuroscience Center Zurich, University of Zurich and ETH Zurich, 8057 Zurich, Switzerland

^13^Zurich Center for Integrative Human Physiology, University of Zurich, 8057 Zurich, Switzerland

†A. Rauch and HU. Zeilhofer are co-senior authors

*corresponding author

**Supplementary Methods**

**Whole exome sequencing**

To re-evaluated the developmental and epileptic encephalopathy (DEE) case published by Kamiya et al. in 2004 carrying the p.R102* variant detected by targeted sequencing of *SCN2A,* we now performed trio whole exome sequencing (WES) of the family using the Agilent (Santa Clara, CA, USA) SureSelect XT Clinical Research Exome Kit (V6) on a HiSeq2500 System (Illumina, San Diego, CA, USA). The WES data was aligned and analyzed using NextGENe Software (SoftGenetics, State College, PA, USA). Average sequencing coverage was at least 20-fold in >96.9% of the exome. Variants were called if observed in at least 16% of reads with sufficient quality level. Rare protein changing variants and splice site variants (including 6 intronic base pairs) with an overall minor allele frequency below 2% were analyzed for de novo calls and, after filtering for known epilepsy or ID genes, for maternally inherited, compound heterozygous, and homozygous calls. Analysis of rare copy number variants was performed from whole exome data using the NxClinical software (BioDiscovery, El Segundo, CA, USA).

**Table S1. Activation and inactivation kinetics of Na_v_1.2 wild-type and mutants**

| Test potential | **10-90% rise time** | | | | **Fast decay time constant** | | | |
| --- | --- | --- | --- | --- | --- | --- | --- | --- |
|  | **wild-type** | **L1342P** | **E1803G** | **L1563V** | **wild-type** | **L1342P** | **E1803G** | **L1563V** |
|  | n=9 | n=9 | n=7 | n=6 | n=9 | n=9 | n=7 | n=6 |
| -30 mV | 1.25 ± 0.13 | 0.57 ± 0.09* | 1.64 ± 0.35 | 1.56 ± 0.27 | 1.63 ± 0.50 | 0.94 ± 0.21 | 14.36 ± 6.41 | 1.96 ± 0.57 |
| -20 mV | 0.78 ± 0.10 | 0.39 ± 0.05 | 1.09 ± 0.24 | 0.90 ± 0.20 | 1.05 ± 0.23 | 0.76 ± 0.10 | 4.82 ± 2.13 | 1.13 ± 0.14 |
| -10 mV | 0.49 ± 0.07 | 0.31 ± 0.03 | 0.73 ± 0.13 | 0.54 ± 0.09 | 0.81 ± 0.14 | 0.57 ± 0.06 | 1.22 ± 0.16 | 0.86 ± 0.11 |
| 0 mV | 0.38 ± 0.05 | 0.26 ± 0.03 | 0.48 ± 0.08 | 0.40 ± 0.06 | 0.58 ± 0.07 | 0.51 ± 0.06 | 0.90 ± 0.11 | 0.66 ± 0.08 |
| 10 mV | 0.31 ± 0.03 | 0.24 ± 0.03 | 0.40 ± 0.06 | 0.32 ± 0.04 | 0.51 ± 0.08 | 0.43 ± 0.06 | 0.77 ± 0.11 | 0.53 ± 0.07 |
| 20 mV | 0.27 ± 0.03 | 0.21 ± 0.02 | 0.33 ± 0.05 | 0.28 ± 0.04 | 0.44 ± 0.06 | 0.40 ± 0.06 | 0.69 ± 0.16 | 0.47 ± 0.05 |
| 30 mV | 0.25 ± 0.02 | 0.19 ± 0.02 | 0.31 ± 0.05 | 0.27 ± 0.03 | 0.40 ± 0.05 | 0.35 ± 0.06 | 0.57 ± 0.13 | 0.40 ± 0.06 |

Values significantly different from wild-type are indicated as follows ∗p < 0.05

**Table S2. Use-dependent inactivation of Na_v_1.2 wild-type and mutants**

| Frequency (Hz) | **Last current relative to first current** | | | |
| --- | --- | --- | --- | --- |
|  | **wild-type** | **L1342P** | **E1803G** | **L1563V** |
|  | n=9 | n=6 | n=7 | n=6 |
| 1 | 0.98 ± 0.02 | 0.99 ± 0.01 | 1.01 ± 0.01 | 1.01 ± 0.01 |
| 10 | 0.91 ± 0.02 | 0.91 ± 0.02 | 0.89 ± 0.05 | 0.94 ± 0.02 |
| 30 | 0.69 ± 0.05 | 0.62 ± 0.05 | 0.64 ± 0.07 | 0.75 ± 0.04 |
| 50 | 0.43 ± 0.07 | 0.34 ± 0.06 | 0.44 ± 0.07 | 0.53 ± 0.08 |
| 70 | 0.20 ± 0.06 | 0.12 ± 0.05 | 0.25 ± 0.03 | 0.28 ± 0.10 |
| 100 | 0.01 ± 0.01 | 0.004 ± 0.004 | 0.02 ± 0.02 | 0.04 ± 0.03 |

**Table S3. Previous electrophysiological studies of disease causing *SCN2A* variants**

| **Publication** | ***SCN2A* variant**  (in neonatal or adult isoform) | **Phenotype**^a^ | **Expression system** | **Overall effect** | **Summarized functional effects of variant on Na_v_1.2 channel** |
| --- | --- | --- | --- | --- | --- |
| Lauxmann et al. 2018 | V208E  T773I  K908E | BFNIE  DEE  BFNIE | tsA201 cells  with β1 and β2 | **GOF**  **GOF**  **GOF** | hyperpolarized activation (by 5mV)  hyperpolarized activation (by 9mV), increased persistent current  increased current density |
| Berecki et al. 2018 | L1563V  R853Q  R1882Q | BFNIE  DEE  DEE | CHO cells  without β subunits | **GOF**  **LOF**  **GOF** | depolarized activation and inactivation (by 1.7 mV / 2.5mV), faster recovery from inactivation  decreased current density (by 110pA/pF), hyperpolarized activation and inactivation (by 1.4mV / 7.4mV)  increased current density (by 162pA/pF), hyperpolarized activation (by 6mV) and depolarized inactivation (by 4.4mV), increased persistent current, slower fast inactivation (*τ*_f_ +0.8ms) |
| Wolff et al. 2017 | V423L  F1597L  G899S  P1622S | DEE  Epilepsy of infancy with migrating focal seizures  DEE  Myoclonic-atonic epilepsy | tsA201 cells  with β1 and β2 | **GOF**  **GOF**  **LOF**  **LOF** | increased persistent current, shallower slope of activation (*K*_a_ +1.5mV)  shallower slope of inactivation (*K*_i_ -1.0mV), slower inactivation (*τ*_f_ +0.41ms), faster recovery from inactivation (*τ*_rec_ -2.7ms)  depolarized activation by 2.2mV  hyperpolarized inactivation by 18.2mV, shallower slope of activation and inactivation (*K*_a_ +0.9mV, *K*_i_ -0.9mV), faster inactivation (*τ*_f_ -0.1ms), slower recovery from inactivation (*τ*_rec_ +4.5ms) |
| Ben-Shalom et al. 2017 | D12N  D82G  T1420M  R379H  S686fs (Ser687Ilefs*33)  R937C  R937H  C959*  G1013*  C1386R | ASD + severe DD + seizures  ASD, no info about DD or seizures  ASD only (no DD, no seizures)  ASD, no info about DD or seizures  ASD, no info about DD or seizures  ASD + severe DD, no seizures  ASD, no info about DD or seizures  ASD, moderate DD  ASD + severe DD  ASD + moderate DD | HEK293 cells  with β1 and β2 | **LOF**  **LOF**  **LOF**  **full LOF**  **full LOF**  **full LOF**  **full LOF**  **full LOF**  **full LOF**  **full LOF** | faster inactivation (*τ* -0.5ms),  smaller peak current, depolarized activation (by 4.9mV)  smaller peak current, faster inactivation (*τ* -0.3ms), reduced surface expression  no conductance  no conductance  no conductance  no conductance  no conductance  no conductance  no conductance |
| Schwarz et al. 2016 | R1882G  R1882G + G1522A | Neonatal-onset seizures, ataxia, myoclonus, pain | tsA201 cells  with β1 and β2 | **GOF**  **GOF** | hyperpolarized activation (by 3.6mV)  depolarized inactivation (by 5.1mV), increased current density (by 302.5pA/pF) |
| Lauxmann et al. 2013 | Y1589C | BFNIE | tsA201 cells  with and without β1 and β2 | **GOF** | depolarized inactivation by 4mV 🡪 increased window current, increased persistent current, slower inactivation (*τ*_f_ +0.1ms), faster recovery from inactivation (*τ*_rec_ -2.0ms), lower peak current density (-458A/F) (numbers from experiments with β subunits) |
| Lossin et al. 2012 | R1312T | DEE | HEK293 cells  with β1 and β2 | **LOF/mixed?** | hyperpolarized activation and inactivation (by 6.8mV / 25.3mV), shallower slope of activation and inactivation (*K*_a_ +1.8mV, *K*_i_ +3.8mV), slower recovery from inactivation (*τ*_rec_ -1.2ms), increased use dependency |
| Liao, Anttonen et al. 2010a | A263V neonatal  A263V adult | Neonatal-onset seizures, ataxia, myoclonus, pain  (this mutation was also reported in two patients with EE (Touma et al. 2013)) | tsA201 cells  with β1 and β2 | **GOF**  **GOF** | increased persistent current, shallower slope of activation and inactivation (*K*_a_ -2.0mV, *K*_i_ +1.3mV) 🡪 increase in window current, slower inactivation (*τ*_f_ +0.11ms), faster recovery from slow inactivation (*τ*_recSI_ -201ms)  increased persistent current, depolarized activation and inactivation (by 6.9mV / 6.7mV), shallower slope of inactivation (*K*_i_ +0.7mV) 🡪 increase in window current, slower inactivation (*τ*_f_ +0.18ms) |
| Liao, Deprez et al. 2010b | M252V neonatal  M252V adult  V261M neonatal  V261M adult | BFNIE | tsA201 cells  with β1 and β2 | **GOF**  **—**  **GOF**  **GOF** | increased persistent current, faster recovery from inactivation (*τ*_f_ -1.4ms)  no significant effect  shallower slope of activation (*K*_a_ -1.6mV), slowed inactivation, faster recovery from inactivation (*τ*_rec_ -1.2ms)  increased persistent current, shallower slope of activation and inactivation (*K*_a_ -1.1mV, *K*_i_ +1.3mV), faster recovery from inactivation (*τ*_rec_ -2.0ms), slower inactivation (*τ*_f_ +0.9ms) |
| Ogiwara et al. 2009 | E1211K  I1473M | DEE | HEK293 cells  with β1 and β2 | **mixed**  **GOF** | hyperpolarized activation and inactivation (by 18.1mV / 22.1mV), slower recovery from inactivation  hyperpolarized activation (by 14.2mV) |
| Misra et al. 2008 | R1319Q  L1330F  L1563V | BFNIE | tsA201 cells  with β1 and β2 | **LOF**  **LOF**  **GOF** | slower recovery from inactivation (*τ*_rec_ +0.3ms), depolarized activation (by 3.9mV), shallower slope of activation (*K*_a_ +1.1mV), slower activation, slower inactivation only at -30mV, lower peak current amplitude  increased use dependency, faster activation only at -10mV, slower inactivation only of slow component (*τ*_s_) and only at few voltage steps  depolarized inactivation (by 4.5mV), slower inactivation, faster recovery from inactivation (*τ*_rec_ -0.4ms), lower peak current amplitude |
| Xu et al. 2007 | L1563V neonatal  L1563V adult | BFNIE | tsA201 cells  without β subunits | **GOF**  **—** | depolarized inactivation by 2.6mV, faster recovery from inactivation, reduced use dependency (20% increased availability)  no significant effect |
| Scalmani et al. 2006 | L1330F  L1563V  R223Q  R1319Q | BFNIE | rat Nav1.2 without β subunits in primary rat neocortical neurons | **GOF**  **GOF**  **GOF**  **GOF** | **on-cell macropatch configuration**  depolarized inactivation by 5.1mV, steeper slope of inactivation (*K*_i_ -0.7mV)  faster activation, hyperpolarized activation by 5.1mV, steeper slope of activation (*K*_a_ -1.5mV)  slower activation and inactivation kinetics, depolarized activation and inactivation (by 4mV / 3.5mV); increased current upon physiological voltage-clamp stimuli  slower activation and inactivation kinetics, depolarized activation and inactivation (by 2.6mV / 3.8mV); increased current upon physiological voltage-clamp stimuli |
| Kamiya et al. 2004 | R102*  R102* + wildtype | ID + ASD + intractable epilepsy  Note that the mother was reported to have febrile seizures and did not carry the mutation | HEK293 cells  with and without β1+β2 | **full LOF**  **LOF** | no conductance  hyperpolarized inactivation (reported as dominant-negative effect); this effect was lost when co-expressed with β-subunits. |
| Sugawara et al. 2001 | R188W | GEFS+ | rat Nav1.2 without β1  in HEK293 cells | **GOF** | slower inactivation, hyperpolarized inactivation by 11.7mV, shallower slope of inactivation curve (*K*_i_ +2.0mV)  **Co-expression of the β1 subunit attenuated the difference in all of these protocols but the data were not shown** |

Abb: ASD autism spectrum disorder; BFNIE self-limited familial neonatal-infantile epilepsy; DD developmental delay; DEE developmental and epileptic encephalopathy; GEFS+ generalized epilepsy with febrile seizures plus; GOF gain of function; ID intellectual disability; *K*_a_ slope factor of activation; *K*_i_ slope factor of inactivation; LOF loss of function; *τ*_rec_ time constant of recovery from inactivation; *τ*_recSI_ time constant of recovery from slow inactivation; *τ*_f_ time constant of fast inactivation; *τ*_s_ time constant of slow inactivation

^a^DEE refers to all types of developmental and/or epileptic encephalopathies.

**Table S4. Previous studies using in-silico modeling of neuronal excitability of disease causing *SCN2A* variants**

| **Publication** | ***SCN2A* variant** | **Phenotype** | **In-silico effect on neuronal excitability** |
| --- | --- | --- | --- |
| Berecki et al. 2018 | L1563V  R853Q  R1882Q | BFNIE  DEE  DEE | Increased action potential firing rate at higher levels of stimulation  Reduced action potential firing rate  Increased action potential firing rate |
| Ben-Shalom et al. 2017 | Non-conducting variants  D12N  D82G  T1420M  L1330F  L1563V  E1211K, I1473M | ASD  ASD + severe DD + seizures  ASD, no info about DD or seizures  ASD only (no DD, no seizures)  BFNIE  BFNIE  DEE, DEE | **All modeled heterozygously with wild-type**  Depolarized spike threshold, reduced spike rate 🡪 hypoexcitability  Depolarized spike threshold, reduced spike rate 🡪 hypoexcitability  Depolarized spike threshold, reduced spike rate 🡪 hypoexcitability  Depolarized spike threshold, reduced spike rate 🡪 hypoexcitability  Hyperpolarized spike threshold, enhanced spike rate 🡪 modest hyperexcitability  Hyperpolarized spike threshold, enhanced spike rate 🡪 modest hyperexcitability  Hyperpolarized spike threshold, increased spike rate, spiking at rest in developmental model 🡪 strong hyperexcitability |
| Xu et al. 2007 | L1563V neonatal  L1563V adult | BFNIE | hyperexcitability  modest hyperexcitability |

Abb: ASD autism spectrum disorder; BFNIE self-limited familial neonatal-infantile epilepsy; DD developmental delay; DEE developmental and epileptic encephalopathy

**Supplementary References**

1. Ben-Shalom R, Keeshen CM, Berrios KN, An JY, Sanders SJ, Bender KJ. Opposing Effects on NaV1.2 Function Underlie Differences Between SCN2A Variants Observed in Individuals With Autism Spectrum Disorder or Infantile Seizures. Biol Psychiatry. 2017;82:224-32.

2. Berecki G, Howell KB, Deerasooriya YH, Cilio MR, Oliva MK, Kaplan D, et al. Dynamic action potential clamp predicts functional separation in mild familial and severe de novo forms of SCN2A epilepsy. Proc Natl Acad Sci U S A. 2018;115:E5516-E25.

3. Kamiya K, Kaneda M, Sugawara T, Mazaki E, Okamura N, Montal M, et al. A nonsense mutation of the sodium channel gene SCN2A in a patient with intractable epilepsy and mental decline. J Neurosci. 2004;24:2690-8.

4. Lauxmann S, Boutry-Kryza N, Rivier C, Mueller S, Hedrich UB, Maljevic S, et al. An SCN2A mutation in a family with infantile seizures from Madagascar reveals an increased subthreshold Na(+) current. Epilepsia. 2013;54:e117-21.

5. Lauxmann S, Verbeek NE, Liu Y, Zaichuk M, Muller S, Lemke JR, et al. Relationship of electrophysiological dysfunction and clinical severity in SCN2A-related epilepsies. Hum Mutat. 2018;39:1942-56.

6. Liao Y, Anttonen AK, Liukkonen E, Gaily E, Maljevic S, Schubert S, et al. SCN2A mutation associated with neonatal epilepsy, late-onset episodic ataxia, myoclonus, and pain. Neurology. 2010a;75:1454-8.

7. Liao Y, Deprez L, Maljevic S, Pitsch J, Claes L, Hristova D, et al. Molecular correlates of age-dependent seizures in an inherited neonatal-infantile epilepsy. Brain. 2010b;133:1403-14.

8. Lossin C, Shi X, Rogawski MA, Hirose S. Compromised function in the Na(v)1.2 Dravet syndrome mutation R1312T. Neurobiol Dis. 2012;47:378-84.

9. Misra SN, Kahlig KM, George AL, Jr. Impaired NaV1.2 function and reduced cell surface expression in benign familial neonatal-infantile seizures. Epilepsia. 2008;49:1535-45.

10. Ogiwara I, Ito K, Sawaishi Y, Osaka H, Mazaki E, Inoue I, et al. De novo mutations of voltage-gated sodium channel alphaII gene SCN2A in intractable epilepsies. Neurology. 2009;73:1046-53.

11. Scalmani P, Rusconi R, Armatura E, Zara F, Avanzini G, Franceschetti S, et al. Effects in neocortical neurons of mutations of the Na(v)1.2 Na+ channel causing benign familial neonatal-infantile seizures. J Neurosci. 2006;26:10100-9.

12. Schwarz N, Hahn A, Bast T, Muller S, Loffler H, Maljevic S, et al. Mutations in the sodium channel gene SCN2A cause neonatal epilepsy with late-onset episodic ataxia. J Neurol. 2016;263:334-43.

13. Sugawara T, Tsurubuchi Y, Agarwala KL, Ito M, Fukuma G, Mazaki-Miyazaki E, et al. A missense mutation of the Na+ channel alpha II subunit gene Na(v)1.2 in a patient with febrile and afebrile seizures causes channel dysfunction. Proc Natl Acad Sci U S A. 2001;98:6384-9.

14. Touma M, Joshi M, Connolly MC, Grant PE, Hansen AR, Khwaja O, et al. Whole genome sequencing identifies SCN2A mutation in monozygotic twins with Ohtahara syndrome and unique neuropathologic findings. Epilepsia. 2013;54:e81-5.

15. Wolff M, Johannesen KM, Hedrich UBS, Masnada S, Rubboli G, Gardella E, et al. Genetic and phenotypic heterogeneity suggest therapeutic implications in SCN2A-related disorders. Brain. 2017;140:1316-36.

16. Xu R, Thomas EA, Jenkins M, Gazina EV, Chiu C, Heron SE, et al. A childhood epilepsy mutation reveals a role for developmentally regulated splicing of a sodium channel. Mol Cell Neurosci. 2007;35:292-301.
